# Gene expression of catabolic inflammatory cytokines peak before anabolic inflammatory cytokines after ACL injury in a preclinical model

**DOI:** 10.1186/s12950-014-0034-3

**Published:** 2014-11-01

**Authors:** Carla M Haslauer, Benedikt L Proffen, Victor M Johnson, Adele Hill, Martha M Murray

**Affiliations:** Department of Orthopaedic Surgery, Boston Children’s Hospital, 300 Longwood Avenue, Boston, MA 02115 USA; Department of Anesthesiology, Boston Children’s Hospital, Boston, MA USA; Department of Genetics, Harvard Medical School, Boston, MA USA

**Keywords:** ACL, Injury, Catabolic, Anabolic, Inflammation, Wound healing, Macrophage, CRP

## Abstract

**Background:**

The response of the joint to anterior cruciate ligament (ACL) injury has not been fully characterized. In particular, the characterization of both catabolic factors, including interleukin-6 (IL-6), interleukin-8 (IL-8), and markers of ongoing tissue damage (CRP), and anabolic factors, including vascular endothelial growth factor (VEGF), transforming growth factor β-induced (TGFβI), and the presence of CD163+ macrophages, have not been well defined. In this study, we hypothesized ACL injury would catalyze both catabolic and anabolic processes and that these would have different temporal profiles of expression.

**Methods:**

Adolescent Yucatan minipigs were subjected to ACL transection. Within the joint, gene expression levels of IL-6, IL-8, VEGF, and TGFβI were quantified in the synovium, ligament, and provisional scaffold located between the torn ligament ends at days 1, 5, 9, and 14 post-injury. Macrophage infiltration was also assessed in the joint tissues over the two week period. Serum C-reactive protein (CRP) levels were measured at multiple time points between 1 hour to 14 days after injury.

**Results:**

Increases in IL-6 and IL-8 gene expression peaked at day 1 after injury in the synovium and ligament. CRP levels were significantly increased at day 3 before returning to pre-injury levels. VEGF and TGFβI gene expression did not significantly increase until day 9 in the synovium and were unchanged in the other tissues. CD163+ macrophages increased in the ligament and synovium until day 9.

**Conclusion:**

Taken together, these results suggest that the response within the joint is primarily catabolic in the first three days after injury, switching to a more anabolic phase by nine days after injury. The effect of medications which alter these processes may thus depend on the timing of administration after injury.

## Introduction

Injury and wound healing are complex processes. The early days after injury are typically marked by an influx of inflammatory cells, such as neutrophils, lymphocytes, and monocytes. The inflammatory cascade is critical in this initial phase of wound healing and facilitates wound debridement and cell recruitment. There are both catabolic and anabolic processes involved in this inflammatory stage. Prolonged catabolism may lead to excessive matrix degradation, while insufficient anabolic responses may lead to a failure of the wound to heal [1]. Previous work in a large animal has shown that a delay of as little as two weeks between anterior cruciate ligament (ACL) injury and enhanced primary repair negatively affects the functional performance of the repaired tissue [2]. The response to injury which develops over time is not yet fully understood, thus there is a need to understand the source of this negative effect as most patients would likely experience a delay between ACL injury and repair.

Prior studies have reported that after injury in ligaments located outside the joint, as in the medial collateral ligament (MCL), an orderly progression of events occurs [3]. There is an initial inflammatory phase which typically lasts up to 5 days [4]. This is then followed by a proliferative phase which can last several weeks after injury [4]. In those extra-articular tissues, the switch from catabolic to anabolic phase is thought to be mediated by the earlier infiltration of macrophages, fibroblasts, and endothelial cells, as deposited provisional extracellular matrix is slowly remodeled [4]. However, while these similar histologic phases have been seen for the ACL after injury [5], it is less well-known how the changes seen in the ligament might be also reflected in the other tissues which may significantly affect the ligament response, namely, synovium and provisional scaffold. We hypothesized that inflammatory changes would be seen not only in the transected ACL, but also in these other important articular structures.

The ACL does not live in isolation; rather, it is in the complex physiologic environment of the synovial fluid, where multiple tissues are metabolically connected by the synovial fluid. The synovial fluid is produced principally by the synovium [6] and bathes the ACL itself, as well as the wound site after ACL transection. To date, the majority of studies evaluating the inflammatory response after ligament injury have studied the ligament or explants of the ligament in isolation [7,8]. For the first time, we are able to look at the simultaneous inflammatory response in both the ligament and the synovium lining the injured joint, both of which have the potential to influence the ligament response after injury. In addition, the blood clot which forms in the joint after injury is another possible source of inflammatory factors, given the relatively high number of leukocytes typically found in that structure in the first days after injury [9]. We have selected synovium, ligament, and this provisional scaffold for examination in this study, as these three tissues in the intact knee are likely to influence the inflammatory response within the knee joint after ACL injury.

In wound healing, the immediate post-injury phase is marked by an early increase in catabolic markers, including C-reactive protein (CRP), interleukin-6 (IL-6) and interleukin-8 (IL-8), which are typically seen within the first few hours to days after an insult or injury [10]. Wound healing then shifts to anabolic functions, with a shift to the reparative, or M2 macrophage phenotype (CD163+), and production of factors that stimulate production of extracellular matrix and revascularization, such as transforming growth factor beta (TGF-β) and vascular endothelial growth factor (VEGF) [11,12]. Recently, there has been interest in stopping or preventing the catabolic response after ACL injury in an effort to slow the early and irreversible loss of cartilage proteins seen after ACL injury [13-15]. However, the optimal timing of this intervention is unknown. We therefore wished to determine when the switch from primarily catabolic to anabolic inflammatory cytokines occurs after ACL injury.

A wide variety of proteins have been identified as markers of early inflammation and wound healing response. IL-6 and IL-8 are pro-inflammatory cytokines which have been shown to be produced during the first 48 hours after an injury, increasing more than 10-fold and 20-fold, respectively, then decreasing over the next 2 weeks in male subjects with an ACL tear [16]. IL-6 and IL-8 are considered important mediators of the acute inflammatory phase of wound healing, appearing in the joint immediately following ACL injury and persisting for approximately 1 week [17]. IL-8 is also considered a main chemoattractant for neutrophils to the wound site, with slow clearance and long-lasting chemoattractant properties in the wound environment [18]. CRP levels are often used as a measure of tissue damage, and elevated levels are observed in patients with ongoing cartilage loss associated with arthritis [19,20]. Knowledge of these early markers of inflammation following ACL injury is of interest in both human patients and animal pathology models, in order to identify new treatment methods and their effects.

In addition to catabolic cytokines, tissue injury stimulates expression of several anabolic factors. Members of the TGF-β superfamily have been found to be key anabolic factors, stimulating collagen production [21] and regulating wound healing, cellular proliferation and differentiation, and angiogenesis [22]. TGFβ-induced (TGFβI) has been detected in various embryonic tissues of the knee during development, including cartilage, meniscal and ligamentous tissues [23]. A lack of TGF-β signaling in the mouse has been shown to negatively affect knee development, resulting in a lack of menisci and cruciate ligaments critical to knee function [23]. VEGF is a known stimulator of angiogenesis and revascularization of tissues [24,25], and expression in the inflammatory phase may contribute to cell migration and proliferation [26]. Increased VEGF expression within the wound site has been correlated with vascular ingrowth, providing a source of nutrients, growth factors and cells to the injured area [25,27]. The timing and source of these markers following ACL injury is of interest for future development of therapeutic strategies.

Macrophages also play a central role in wound healing, accumulating at the site of injury and performing a wide variety of tasks, ranging from phagocytosis of debris to scar tissue formation and regulation of angiogenesis [28]. Recent work has attempted to identify and classify macrophages along a spectrum determined by their means of activation, as well as their function. CD68 is highly expressed by monocytes and macrophages and is commonly used to indicate undifferentiated macrophages [29,30]. After a period of time within the wound, macrophages, in particular, change to an alternatively activated, anabolic phenotype (CD163+ or M2). CD163+ macrophages release anabolic cytokines and secrete extracellular matrix (ECM) proteins, thereby playing a more positive role in the wound healing response [28,29].

In this study, we hypothesized that ACL injury would result in an increase in early catabolic markers, particularly CRP, IL-6, and IL-8, in the first few days after injury. We also hypothesized than an increase in anabolic markers of the proliferative phase would be observed later in the two week period.

## Materials and methods

Thirty adolescent Yucatan minipigs (Coyote CCI, Douglas, MA), aged 12–15 months, were obtained for use in this study. All minipigs were handled according to approved IACUC protocols at Animal Resources at Children’s Hospital (ARCH, Boston, MA). Twenty-four minipigs were subjected to ACL transection, followed by tissue harvest at 1, 5, 9, or 14 days post-injury. A group of 6 Yucatans was used to provide unoperated control specimens.

### Surgical procedure

Unilateral ACL transection was performed as previously described [31,32]. Briefly, the ACL was exposed by performing a medial arthrotomy and partial resection of the fat pad. The ACL was cut using a scalpel blade at the junction of the proximal and middle thirds, functional loss of the ACL verified, and the knee closed in layers. Minipigs were allowed normal nutrition and *ad lib* activity following surgery throughout the experimental period.

### Tissue collection

The ACL, including any provisional scaffold matrix found on the end of the injured ligament, and medial synovium from each minipig were harvested after the specified time points from the injured knee of each subject (day 1, 5, 9, or 14, n = 6 for each time point) as previously described [32]. Each tissue specimen was submerged in a cryovial containing RNALater (Ambion, Austin, TX, USA), flash frozen in liquid nitrogen, then stored at −80°C until analyses. A portion of the ligament tissue was also embedded within OCT medium (Sakura Finetek, CA, USA), frozen, and stored at −80°C for histological analysis. Synovium and intact ACL tissue were also harvested from six control subjects. Systemic blood of control minipigs was clotted to serve as a provisional scaffold control for the intact ACL group.

The animals belonging to the groups sacrificed at day 9 and 14 were also subjected to serum draws at alternating time points. IACUC protocols demanded a break between anesthesia events. Therefore, one group was sampled pre-transection, then 1 h, 5 days, 12 days, and 14 days post-injury, while a second group was sampled at 3 h, 1 day, 3 days, 7 days and 9 days post-injury. Blood was collected in serum separator tubes, clotted at room temperature, centrifuged at 1000X g for 10 min, and the serum aliquoted in 500 μL aliquots and stored at −80°C.

### C-reactive protein

CRP concentrations in serum aliquots were quantified via the Pig C-Reactive Protein ELISA Test Kit (Life Diagnostics, Inc., West Chester, PA) according to the manufacturer’s protocol.

### qPCR and data analysis

The ligament, synovium, and provisional scaffold were examined for mRNA expression of several genes using real-time reverse transcriptase polymerase chain reaction (qPCR) run in duplicate as previously described [32]. Briefly, total RNA was extracted from frozen tissue, treated with DNAse I, quantified, and reverse transcribed to generate cDNA. Primers are summarized in Table 1. Sybr Green PCR Mastermix (Applied Biosystems, Foster City, CA, USA), water, forward and reverse primer, and 10 ng cDNA were mixed and quantified in a reaction volume of 10 μl. No template controls were included to indicate contaminants or non-specific amplification and an Applied Biosystems 7900HT (Applied Biosystems) was used for amplification and detection. Level of gene expression was normalized to the housekeeping gene, GAPDH. Relative gene expression was calculated using the 2^-ΔCt^ method [33].

**Table 1:** **Sequences of porcine-specific qPCR primers**

**Gene**	**Forward primer**	**Reverse primer**
**GAPDH [** 34 **]**	GGG CAT GAA CCA TGA GAA GT	GTC TTC TGG GTG GCA GTG AT
**IL-6 [** 35 **]**	TGG GTT CAA TCA GGA GAC CT	CAG CCT CGA CAT TTC CCT TA
**IL-8 [** 35 **]**	GAA GAG AAC TGA GAA GCA ACA ACA	TTG TGT TGG CAT CTT TAC TGA GA
**VEGF**	ATC TTC AAG CCG TCC TGT GT	TGC ATT CAC ATT TGT TGT GC
**TGFβI [** 23 **]**	ATC GGA ATA GCC TGT GCA TC	CCT GGA AGG CTT CAT TGG TA

### Macrophage histology

Ligament, synovium and provisional scaffold tissue specimens were sectioned (6 μm) and individually stained with CD163 (MCA2311, AbD Serotec, Raleigh, NC) and counterstained with hematoxylin (Mass Histology Service, Inc., Worcester, MA). No pre-treatment was required, and tissue sections were incubated in the primary antibody at a 1:250 dilution overnight at 4°C. The sections were then incubated for 45 min in the secondary antibody (Vector mouse ImmPRESS kit, Vector Laboratories, Burlingame, CA) followed by custom labeling with DAB for 1 minute.

### Statistical analyses

The time course of serum CRP levels was described using repeated-measures analysis of variance with an autoregressive covariance structure (PROC MIXED, SAS version 9.3, SAS Institute, Cary, NC). Gene expression was summarized as gene expression per cell, mean and standard deviation. Overall differences in gene expression levels (both across time for a particular gene and within each tissue) were assessed using a two-factor ANOVA followed by post-hoc Bonferroni-Dunn testing and one-way ANOVA to assess the effect of time on the gene expression in the individual tissues. P < 0.05 was considered statistically significant for all analyses. Statistical analyses were performed using SAS (version 9.2, SAS Institute Inc., Cary, NC) and Statview (version 5.0.1, SAS Institute Inc.).

## Results

### Catabolic factors

#### C-reactive protein

**Figure 1:**
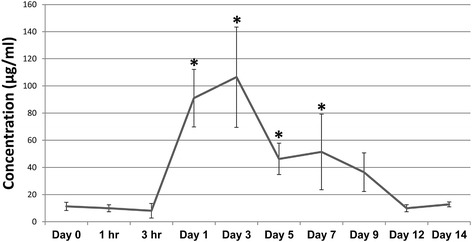
**CRP concentrations in serum collected pre- and post-injury.** Data represent mean concentration ± SEM. Asterisks indicate significance determined at P < 0.05 compared to baseline (Day 0) concentrations.

Serum CRP levels increased following ACL injury (Figure 1), with a significant increase (5-fold) over baseline observed at day 1 (P < 0.0001). CRP levels continued to rise and were significantly elevated (6-fold) over baseline at day 3 (P < 0.0001), then slowly returned to normal levels over the remainder of the two week period though significant increases over baseline were still observed at days 5 and 7 (P = 0.0361 and 0.0133, respectively).

#### IL-6 gene expression

**Figure 2:**
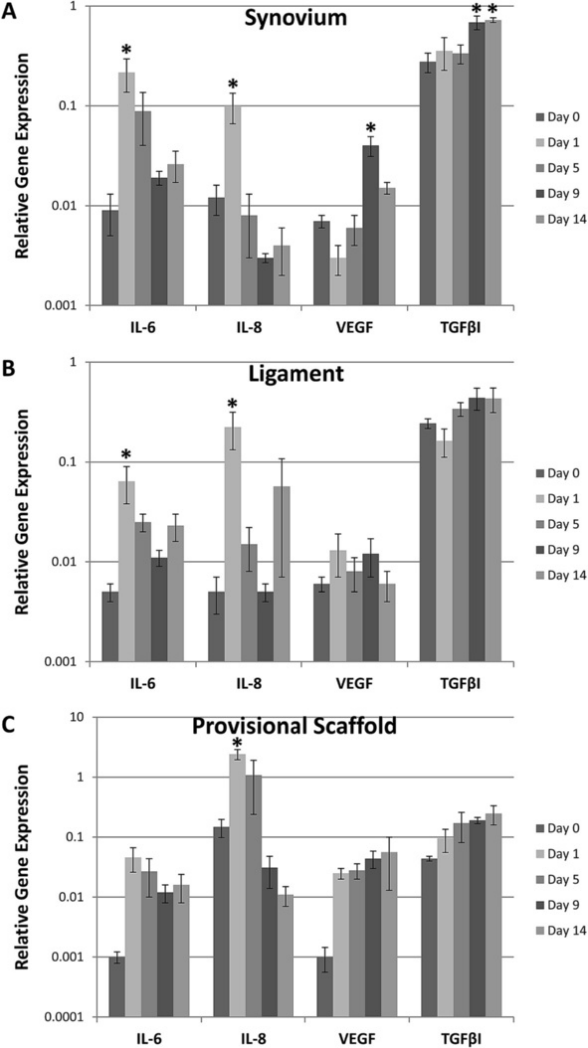
**Mean gene expression relative to GAPDH of IL-6, IL-8, VEGF, and TGFβI in the (A) synovium, (B) ligament, and (C) provisional scaffold following ACL injury.** Note the log 10 scale on the y-axis. Error bars represent standard error of the mean and asterisks indicate significance determined at P < 0.05 within tissue type compared to baseline (Day 0) levels.

Expression of IL-6 was significantly higher in the synovium when compared to the ligament and provisional scaffold (P < 0.01 for both comparisons) and peaked at day 1 after injury (P < 0.0001) where the levels of IL-6 gene expression in the synovium had increased by 20X over baseline (P < 0.001) (Figure 2A). The ligament had a 12-fold increase in IL-6 gene expression from day 0 to day 1 (P < 0.003) (Figure 2B) and the scaffold had a 46-fold increase in expression from day 0 to day 1 (P < 0.02, not significant) (Figure 2C). After the peak at day 1, levels in all tissues had significantly decreased by day 9 (P < 0.0001).

#### IL-8 gene expression

IL-8 gene expression levels were significantly higher in the provisional scaffold when compared to the ligament and synovium (P < 0.0001 for both comparisons) and peaked at day 1 for all tissues (P < 0.0001). There the levels of expression were 10X higher in the provisional scaffold than in the synovium (Figure 2C). The provisional scaffold had a 16-fold increase in expression from day 0 to day 1 (P < 0.0001), while the ligament had a 44- fold increase in expression from day zero (P < 0.0025) (Figure 2B) and the synovium had an 8-fold increase in expression from day zero to day 1 (P < 0.001) (Figure 2A). Levels in all tissues had significantly decreased by day 9 (P < 0.0001).

### Anabolic factors

#### VEGF gene expression

A significant increase in VEGF gene expression from baseline levels was noted in the synovium at day 9 (P < 0.0001). VEGF gene expression levels were significantly higher in the provisional scaffold when compared to the ligament (P < 0.008), but not significantly different from the synovium (P < 0.04). On average, the gene expression levels of VEGF were 2.5X higher in the provisional scaffold than in the ligament (Figure 2).

#### TGFβI gene expression

TGFβI expression levels significantly increased in all tissues over the course of the experiment (Figure 2, P < 0.0001 for all comparisons between day 0 and days 9 and 14, as well as between day 1 and days 9 and 14). TGFβI gene expression levels were significantly higher in the synovium when compared to the ligament and provisional scaffold (P < 0.001 for both comparisons) and were higher in the ligament than in the provisional scaffold (P < 0.0008).

#### CD163+ macrophages

**Figure 3:**
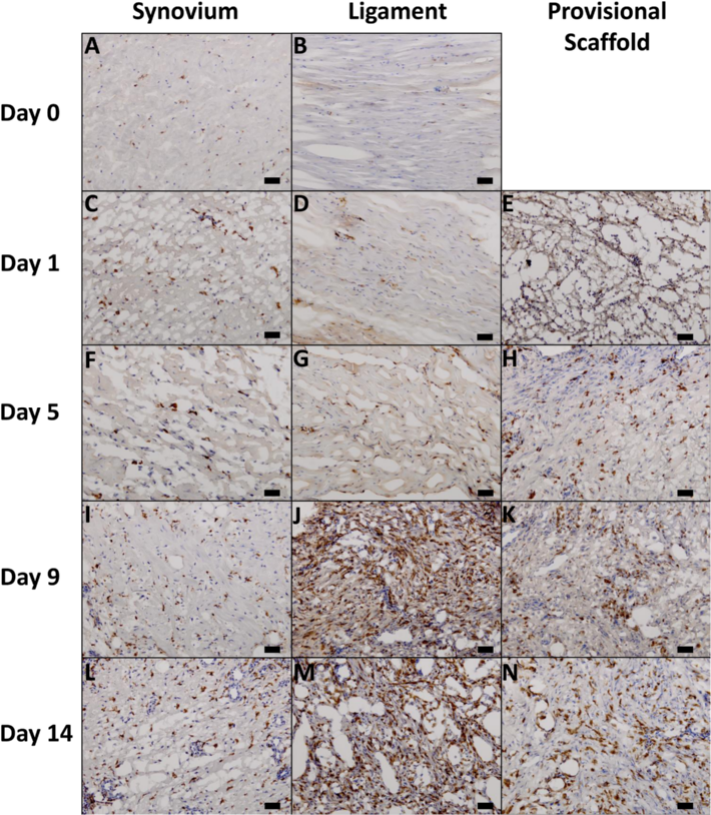
**Light microscopy images of CD163 antibody stained histological sections obtained from the synovium (A, C, F, I, L), ligament (B, D, G, J, M), and provisional scaffold (E, H, K, N) at days 0 (A,B), 1 (C, D, E), 5 (F, G, H), 9 (I, J, K), and 14 (L, M, N) following ACL injury. Scale bar represents 50 μm.**

In the intact ligament, very few CD163+ cells were seen (Figure 3B). Following ACL injury, CD163+ cells increased in number within the ligament, as well as within the synovium and provisional scaffold. The highest concentrations of CD163+ cells were observed at days 9 (Figure 3I, J, and K) and 14 in the synovium, ligament, and provisional scaffold (Figure 3L, M, and N).

## Discussion

In the ACL injury model, even without direct injury to the synovium in the areas evaluated, an initial catabolic response was seen in both the ligament and synovium. Specifically, IL-6 and IL-8 were significantly upregulated at day 1, returning toward normal levels by day 5. This was reflected in the systemic blood, as well with an increase in CRP levels in the plasma peaking at day 3. Taken together, these changes suggest that an ACL transection results in an immediate coordinated catabolic response by the synovium as well as the ligament. While there were no significant indicators of anabolic function in the ligament tissue, the synovium had significant increases in the gene expression of both VEGF (day 9) and TGFβI (days 9 and 14) later in the time course, suggesting that while a productive response to injury may be occurring in the synovium, it is less clear this occurs in the transected, intra-articular ACL. This would be consistent with prior reports of the failure of the transected ACL to mount an effective healing response [5,36].

Following ACL transection, CRP levels peaked at 3 days following injury, exhibiting a strong correlation to human patients undergoing ACL reconstruction, where mean CRP levels increased significantly the first post-operative day, peaked on day 3, and returned to baseline levels by day 15 [37]. Similar results were also observed in the plasma of human patients undergoing ACL reconstruction, with significantly increased plasma CRP levels noted 3 days after the operation, which returned to preoperative levels by 2 weeks post-surgery [38]. In addition, these data suggest the porcine model has a similar CRP response to the human knee, and therefore may be a reasonable surrogate for studying human joint injury.

There are multiple candidate theories for the observed switch from catabolic to anabolic processes within the joint after ACL transection. Here, IL-6 and IL-8 gene expression levels were both seen to peak at day one in both the ligament and synovium before declining, which is consistent with reports of human synovial fluid levels following ACL injury [17]. IL-8 in particular is released by neutrophils in the wound site. These cells undergo apoptosis within 1 to 3 days, and engulfment of the apoptotic neutrophils can change the wound macrophages from a catabolic phenotype to an anabolic phenotype (CD163+), where they release VEGF [39]. Indeed in this study, we see an increase in VEGF expression within the synovial tissue at day 9, as well as an increase in CD163+ macrophages, particularly within the wounded ligament at this same time point. Thus, the IL-8/neutrophil/macrophage axis may be particularly relevant in this injury model.

These results correlate with similar studies of synovial fluid human patients where a peak in IL-6 levels was observed within 24 hours after ACL injury and an increase in IL-8 levels was apparent within 3 days of injury before declining [17]. Interestingly, persistent presence of higher levels of IL-6 in the synovial fluid was correlated with painful ACL injuries; non-painful knees did not have elevated levels of IL-6 [40]. Increased interferon gamma and macrophage inflammatory protein beta concentrations were also observed in the synovial fluid of these injured patients [40]. Another study in humans with ACL tears similarly observed elevated IL-6 and IL-8 levels in the synovial fluid initially, which decreased in concentration rapidly within 3 days of injury [17]. A similar peak was also seen in tumor necrosis factor alpha (TNF-α), IL-1β, and interleukin-1 receptor antagonist (IL-1RA) concentrations in these patients, however, TNF-α levels remained relatively high through 2 weeks and IL-1β and IL-1RA were high until 6 days after injury [17].

Previous work by Nakahara et al. indicated that IL-6 induces VEGF production in human synovial fibroblasts [41]. In this study, IL-6 gene expression levels peaked at day 1, followed by a significant increase in VEGF gene expression levels at day 9 in the synovium. VEGF concentrations in the drain fluid of patients undergoing ACL reconstruction were also found to significantly increase with time from surgery [42]. In studies of reconstructed ACL graft tissue, the highest vascularity was observed between 6–12 weeks [43,44]. In transected ACLs treated with a suture repair plus a collagen-platelet composite sponge, blood vessel concentration at the insertion site was greatly increased at both 2 and 4 weeks in the adolescent injury model [45]. Thus, the initial inflammatory response may elicit VEGF gene expression and result in increased angiogenesis within the native ligament tissue.

This study focuses on changes within the first two weeks of ACL injury, though other studies have evaluated the changes in gene expression in joint tissues at longer time points [46,47]. Naraoka et al. found increased IL-6 gene expression within the first three weeks after injury in human ACL tissue, as well as an increase in Collagen I gene expression in the 3–8 week timeframe [47]. Additionally, in a 6 week study in rabbits, Attia et al. noted increased MMP-13 gene expression in the ligament at 1 week, with increased Collagen I and III gene expression at the 6 week time point [46]. However, these studies often evaluated a single tissue rather than three joint tissues, the ligament, synovium, and provisional scaffold, simultaneously. Future studies to evaluate the simultaneous joint changes at longer time points are planned.

TGFβI expression was found to increase in all joint tissues after ACL injury, with a peak increase seen at 9 days after injury. In prior studies, mRNA levels of TGFβI have been shown to increase following macrophage engulfment of apoptotic cells [48]. Dose and time effects of TGFβI treatment were also shown to regulate collagen levels in cultured fibroblasts, suggesting regulation may occur through excretion of soluble factors by the macrophages [48]. These results suggest that TGFβI may act as a mediator of the pro-fibrotic effects exhibited by macrophages following ingestion of apoptotic cells [48]. Previous analyses from ACL injured and normal patients have also shown a significant increase in both type I and type III collagen gene expression in injured patients compared to normal expression in the ligament [49]. Furthermore, in a study of patients undergoing ACL reconstruction, TGF-β and platelet-derived growth factor (PDGF), which promotes cell migration and plays a significant role in angiogenesis, were found to rapidly increase in protein concentration in the drain fluid within 6 hours of surgery [42]. These increases in anabolic proteins indicate there is a potential to mount a scarring response followed by healing after ACL injury, however, the resulting non-functional scar may be due to the early increase in catabolic proteins.

The presence of CD163+ macrophages was found to increase after injury, plateauing at day 9. In other wound healing models, macrophages have been found to migrate from the peripheral blood toward a gradient of chemoattractants, eventually accumulating at the wound site. The CD163+ macrophages have previously been associated with anabolic wound healing activities, including stimulating increased collagen production in fibroblasts following ingestion of apoptotic debris [29,48]. The CD163+ phenotype has been described as the hallmark of the wound healing macrophage, dampening the effects of oxidative injury from hemolysis during bruising or tissue injury, and transitioning into this “resolving macrophage” phenotype with increasing time from injury- even in less traumatic skin blistering models [50]. Thus, their presence in the later phase of the response to ACL injury may be another marker of the shift from catabolic to anabolic processes in the joint.

The early biological changes which occur following ACL injury are believed to play an important role in subsequent joint health. In addition to the ligament’s limited capacity to heal without surgical intervention, which largely serves to impart mechanical stability to the injured joint, post-traumatic osteoarthritis has been shown to develop within 10–20 years in more than 50% of individuals who experience an ACL tear [51]. Loss of proteoglycan and collagen molecules from cartilage has been shown within the first few weeks after injury [52,53]. Neuman et al. observed evidence of reduced glycosaminoglycan content in cartilage from patients examined at 3 weeks and 2.3 years after ACL injury [54]. We have similarly shown an increase of type II collagen fragments in synovial fluid, which nearly doubled within 5 days of ACL injury [32]. Thus, knowledge of, and intervention in, the catabolic pathways initiated by ACL rupture may help prevent further damage to both the ligament and cartilaginous structures after injury.

A few study limitations should be noted. Most importantly, an animal model was required to allow for tissue harvest at various time points after injury. Thus, it is possible that the timing of CRP production, cytokine gene expression, and macrophage infiltration may be different in humans; however, the CRP [37] and IL-6 [17] profiles reported here closely matches that previously reported for humans with ACL injury. The porcine model was chosen in part due to its size, ACL dependency, and gait similarity to humans [55-58]. There is extensively conserved homology between the porcine genome and the human genome, far more than between human and mouse or other rodents [59]. The pig is therefore an important model for human health, particularly for understanding complex traits such as wound healing, obesity, and cardiovascular disease [60-62]. In addition, the porcine knee is approximately the same size as the knee of a small human, thus diseases which may be dependent on tissue size are more easily modeled in this organism, as has already been shown for retinitis and diabetes [63,64]. This model has been used to study ACL injury and surgical and tissue engineering approaches for healing, thus allowing for comparisons and further insight into the biological mechanisms following injury [65-67]. The minipig also serves as a model for other human conditions, including testing of diabetes drugs and mechanical heart valves, and even serves as a source of donor tissue for prosthetic aortic valves [68-70]. Additionally, mRNA expression is reported here, however, expression levels may not directly correlate with protein release. Another limitation was the control tissue specimens were obtained from a separate group of animals as altered gait due to ACL injury could potentially affect the tissues of the contralateral knee.

## Conclusions

ACL injury, similar to other wound responses, has both a catabolic and anabolic response. In this study, we report that while the catabolic factors evaluated in this study are active primarily in the first three days after injury, the anabolic factors studied here become more prominent at a week or more from injury. Future studies aim to regulate these processes, with the goal of minimizing the catabolic phase of tissue destruction and accelerating the anabolic phase of tissue repair.

## Abbreviations

ACL: Anterior cruciate ligament
CRP: C-reactive protein
IL-6: Interleukin-6
IL-8: Interleukin-8
VEGF: Vascular endothelial growth factor
TGFβI: Transforming growth factor β-induced
ECM: Extracellular matrix
qPCR: Real-time reverse transcriptase polymerase chain reaction

## References

[1] Midwood KS, Williams LV, Schwarzbauer JE (2004). Tissue repair and the dynamics of the extracellular matrix. Int J Biochem Cell Biol.

[2] Magarian EM, Fleming BC, Harrison SL, Mastrangelo AN, Badger GJ, Murray MM (2010). Delay of 2 or 6 weeks adversely affects the functional outcome of augmented primary repair of the porcine anterior cruciate ligament. Am J Sports Med.

[3] Frank C, Schachar N, Dittrich D (1983). Natural history of healing in the repaired medial collateral ligament. J Orthop Res.

[4] Chamberlain CS, Crowley E, Vanderby R (2009). The spatio-temporal dynamics of ligament healing. Wound Repair Regen.

[5] Murray MM, Martin SD, Martin TL, Spector M (2000). Histological changes in the human anterior cruciate ligament after rupture. J Bone Joint Surg Am.

[6] Hui AY, McCarty WJ, Masuda K, Firestein GS, Sah RL (2012). A systems biology approach to synovial joint lubrication in health, injury, and disease. Wiley Interdiscip Rev Syst Biol Med.

[7] Binks DA, Gravallese EM, Bergin D, Hodgson RJ, Tan AL, Matzelle MM, McGonagle D, Radjenovic A (2013). Role of vascular channels as a novel mechanism for subchondral bone damage at cruciate ligament entheses in osteoarthritis and inflammatory arthritis. Ann Rheum Dis.

[8] Heard BJ, Solbak NM, Achari Y, Chung M, Hart DA, Shrive NG, Frank CB (2013). Changes of early post-traumatic osteoarthritis in an ovine model of simulated ACL reconstruction are associated with transient acute post-injury synovial inflammation and tissue catabolism. Osteoarthritis Cartilage/Osteoarthritis Res Soc.

[9] Gillitzer R, Goebeler M (2001). Chemokines in cutaneous wound healing. J Leukoc Biol.

[10] Bryan D, Walker KB, Ferguson M, Thorpe R (2005). Cytokine gene expression in a murine wound healing model. Cytokine.

[11] Diegelmann RF, Evans MC (2004). Wound healing: an overview of acute, fibrotic and delayed healing. Front Biosci.

[12] Wolfs IM, Donners MM, de Winther MP (2011). Differentiation factors and cytokines in the atherosclerotic plaque micro-environment as a trigger for macrophage polarisation. Thromb Haemost.

[13] Pickarski M, Hayami T, Zhuo Y, Duong le T (2011). Molecular changes in articular cartilage and subchondral bone in the rat anterior cruciate ligament transection and meniscectomized models of osteoarthritis. BMC Musculoskelet Disord.

[14] Wu H, Du J, Zheng Q (2008). Expression of MMP-1 in cartilage and synovium of experimentally induced rabbit ACLT traumatic osteoarthritis: immunohistochemical study. Rheumatol Int.

[15] Teeple E, Elsaid KA, Fleming BC, Jay GD, Aslani K, Crisco JJ, Mechrefe AP (2008). Coefficients of friction, lubricin, and cartilage damage in the anterior cruciate ligament-deficient guinea pig knee. J Orthop Res.

[16] Bigoni M, Sacerdote P, Turati M, Franchi S, Gandolla M, Gaddi D, Moretti S, Munegato D, Augusti CA, Bresciani E, Omeljaniuk RJ, Locatelli V, Torsello A (2012). Acute and late changes in intraarticular cytokine levels following anterior cruciate ligament injury. J Orthop Res.

[17] Irie K, Uchiyama E, Iwaso H (2003). Intraarticular inflammatory cytokines in acute anterior cruciate ligament injured knee. Knee.

[18] Baggiolini M, Clark-Lewis I (1992). Interleukin-8, a chemotactic and inflammatory cytokine. FEBS Lett.

[19] Liao H, Wu J, Kuhn E, Chin W, Chang B, Jones MD, O’Neil S, Clauser KR, Karl J, Hasler F, Roubenoff R, Zolg W, Guild BC (2004). Use of mass spectrometry to identify protein biomarkers of disease severity in the synovial fluid and serum of patients with rheumatoid arthritis. Arthritis Rheum.

[20] Wassilew GI, Lehnigk U, Duda GN, Taylor WR, Matziolis G, Dynybil C (2010). The expression of proinflammatory cytokines and matrix metalloproteinases in the synovial membranes of patients with osteoarthritis compared with traumatic knee disorders. Arthroscopy.

[21] Chen SJ, Yuan W, Mori Y, Levenson A, Trojanowska M, Varga J (1999). Stimulation of type I collagen transcription in human skin fibroblasts by TGF-beta: involvement of Smad 3. J Invest Dermatol.

[22] Blobe GC, Schiemann WP, Lodish HF (2000). Role of transforming growth factor beta in human disease. N Engl J Med.

[23] Pazin DE, Gamer LW, Cox KA, Rosen V (2012). Molecular profiling of synovial joints: Use of microarray analysis to identify factors that direct the development of the knee and elbow. Dev Dyn.

[24] Jackson JR, Minton JA, Ho ML, Wei N, Winkler JD (1997). Expression of vascular endothelial growth factor in synovial fibroblasts is induced by hypoxia and interleukin 1beta. J Rheumatol.

[25] Gelberman RH, Khabie V, Cahill CJ (1991). The revascularization of healing flexor tendons in the digital sheath. A vascular injection study in dogs. J Bone Joint Surg Am.

[26] Molloy T, Wang Y, Murrell G (2003). The roles of growth factors in tendon and ligament healing. Sports Med.

[27] Boyer MI, Watson JT, Lou J, Manske PR, Gelberman RH, Cai SR (2001). Quantitative variation in vascular endothelial growth factor mRNA expression during early flexor tendon healing: an investigation in a canine model. J Orthop Res.

[28] Chamberlain CS, Leiferman EM, Frisch KE, Wang S, Yang X, van Rooijen N, Baer GS, Brickson SL, Vanderby R (2011). The influence of macrophage depletion on ligament healing. Connect Tissue Res.

[29] Badylak SF, Valentin JE, Ravindra AK, McCabe GP, Stewart-Akers AM (2008). Macrophage phenotype as a determinant of biologic scaffold remodeling. Tissue Eng A.

[30] Holness CL, Simmons DL (1993). Molecular cloning of CD68, a human macrophage marker related to lysosomal glycoproteins. Blood.

[31] Murray MM, Magarian E, Harrison SL, Mastrangelo A, Zurakowski D, Fleming BC (2010). The effect of skeletal maturity on functional healing of the anterior cruciate ligament. J Bone Joint Surg.

[32] Haslauer CM, Elsaid KA, Fleming BC, Proffen BL, Johnson VM, Murray MM (2013). Loss of extracellular matrix from articular cartilage is mediated by the synovium and ligament after anterior cruciate ligament injury. Osteoarthritis Cartilage.

[33] Schmittgen TD, Livak KJ (2008). Analyzing real-time PCR data by the comparative C(T) method. Nat Protoc.

[34] Cheng M, Wang H, Yoshida R, Murray MM (2010). Platelets and plasma proteins are both required to stimulate collagen gene expression by anterior cruciate ligament cells in three-dimensional culture. Tissue Eng Part A.

[35] Skovgaard K, Mortensen S, Boye M, Hedegaard J, Heegaard PM (2010). Hepatic gene expression changes in pigs experimentally infected with the lung pathogen Actinobacillus pleuropneumoniae as analysed with an innate immunity focused microarray. Innate Immun.

[36] Hefti FL, Kress A, Fasel J, Morscher EW (1991). Healing of the transected anterior cruciate ligament in the rabbit. J Bone Joint Surg Am.

[37] Calvisi V, Lupparelli S (2008). C-reactive protein changes in the uncomplicated course of arthroscopic anterior cruciate ligament reconstruction. Int J Immunopathol Pharmacol.

[38] Mendias CL, Lynch EB, Davis ME, Sibilsky Enselman ER, Harning JA, Dewolf PD, Makki TA, Bedi A (2013). Changes in circulating biomarkers of muscle atrophy, inflammation, and cartilage turnover in patients undergoing anterior cruciate ligament reconstruction and rehabilitation. Am J Sports Med.

[39] Golpon HA, Fadok VA, Taraseviciene-Stewart L, Scerbavicius R, Sauer C, Welte T, Henson PM, Voelkel NF (2004). Life after corpse engulfment: phagocytosis of apoptotic cells leads to VEGF secretion and cell growth. FASEB J.

[40] Cuellar VG, Cuellar JM, Golish SR, Yeomans DC, Scuderi GJ (2010). Cytokine profiling in acute anterior cruciate ligament injury. Arthroscopy.

[41] Nakahara H, Song J, Sugimoto M, Hagihara K, Kishimoto T, Yoshizaki K, Nishimoto N (2003). Anti-interleukin-6 receptor antibody therapy reduces vascular endothelial growth factor production in rheumatoid arthritis. Arthritis Rheum.

[42] Hayward AL, Deehan DJ, Aspden RM, Sutherland AG (2011). Analysis of sequential cytokine release after ACL reconstruction. Knee Surg, Sports Traumatol, Arthrosc.

[43] Meller R, Brandes G, Drogemuller C, Fritz F, Schiborra F, Fehr M, Hankemeier S, Krettek C, Hurschler C (2009). Graft remodeling during growth following anterior cruciate ligament reconstruction in skeletally immature sheep. Arch Orthop Trauma Surg.

[44] Weiler A, Unterhauser FN, Bail HJ, Huning M, Haas NP (2002). Alpha-smooth muscle actin is expressed by fibroblastic cells of the ovine anterior cruciate ligament and its free tendon graft during remodeling. J Orthop Res.

[45] Haus BM, Mastrangelo AN, Murray MM (2012). Effect of anterior cruciate healing on the uninjured ligament insertion site. J Orthop Res.

[46] Attia E, Brown H, Henshaw R, George S, Hannafin JA (2010). Patterns of gene expression in a rabbit partial anterior cruciate ligament transection model: the potential role of mechanical forces. Am J Sports Med.

[47] Naraoka T, Ishibashi Y, Tsuda E, Yamamoto Y, Kusumi T, Kakizaki I, Toh S (2012). Time-dependent gene expression and immunohistochemical analysis of the injured anterior cruciate ligament. Bone Joint Res.

[48] Nacu N, Luzina IG, Highsmith K, Lockatell V, Pochetuhen K, Cooper ZA, Gillmeister MP, Todd NW, Atamas SP (2008). Macrophages produce TGF-beta-induced (beta-ig-h3) following ingestion of apoptotic cells and regulate MMP14 levels and collagen turnover in fibroblasts. J Immunol.

[49] Lo IK, Marchuk LL, Hart DA, Frank CB (1998). Comparison of mRNA levels for matrix molecules in normal and disrupted human anterior cruciate ligaments using reverse transcription-polymerase chain reaction. J Orthop Res.

[50] Evans BJ, Haskard DO, Sempowksi G, Landis RC (2013). Evolution of the macrophage CD163 phenotype and cytokine profiles in a human model of resolving inflammation. Int J Inflam.

[51] Lohmander LS, Englund PM, Dahl LL, Roos EM (2007). The long-term consequence of anterior cruciate ligament and meniscus injuries: osteoarthritis. Am J Sports Med.

[52] Fleming BC, Oksendahl HL, Mehan WA, Portnoy R, Fadale PD, Hulstyn MJ, Bowers ME, Machan JT, Tung GA (2010). Delayed Gadolinium-Enhanced MR Imaging of Cartilage (dGEMRIC) following ACL injury. Osteoarthritis Cartilage/Osteoarthritis Res Soc.

[53] Catterall JB, Stabler TV, Flannery CR, Kraus VB (2010). Changes in serum and synovial fluid biomarkers after acute injury (NCT00332254). Arthritis Res Ther.

[54] Neuman P, Tjornstrand J, Svensson J, Ragnarsson C, Roos H, Englund M, Tiderius CJ, Dahlberg LE (2011). Longitudinal assessment of femoral knee cartilage quality using contrast enhanced MRI (dGEMRIC) in patients with anterior cruciate ligament injury–comparison with asymptomatic volunteers. Osteoarthritis Cartilage/Osteoarthritis Res Soc.

[55] Andriacchi TP, Briant PL, Bevill SL, Koo S (2006). Rotational changes at the knee after ACL injury cause cartilage thinning. Clin Orthop Relat Res.

[56] Levy AS, Meier SW (2003). Approach to cartilage injury in the anterior cruciate ligament-deficient knee. Orthop Clin North Am.

[57] Sharma L, Lou C, Felson DT, Dunlop DD, Kirwan-Mellis G, Hayes KW, Weinrach D, Buchanan TS (1999). Laxity in healthy and osteoarthritic knees. Arthritis Rheum.

[58] Seitz H, Hausner T, Schlenz I, Lang S, Eschberger J (1997). Vascular anatomy of the ovine anterior cruciate ligament. A macroscopic, histological and radiographic study. Arch Orthop Trauma Surg.

[59] Humphray SJ, Scott CE, Clark R, Marron B, Bender C, Camm N, Davis J, Jenks A, Noon A, Patel M, Sehra H, Yang F, Rogatcheva MB, Milan D, Chardon P, Rohrer G, Nonneman D, de Jong P, Meyers SN, Archibald A, Beever JE, Schook LB, Rogers J (2007). A high utility integrated map of the pig genome. Genome Biol.

[60] Kilpadi DV, Lessing C, Derrick K (2014). Healed porcine incisions previously treated with a surgical incision management system: mechanical, histomorphometric, and gene expression properties. Aesthet Plast Surg.

[61] Gonzalez-Bulnes A, Astiz S, Sanchez-Sanchez R, Perez-Solana M, Gomez-Fidalgo E (2013). Maternal diet-induced obesity in swine with leptin resistance modifies puberty and pregnancy outputs of the adult offspring. J Dev Orig Health Dis.

[62] Behler RH, Czernuszewicz TJ, Wu CD, Nichols TC, Zhu H, Homeister JW, Merricks EP, Gallippi CM (2013). Acoustic radiation force beam sequence performance for detection and material characterization of atherosclerotic plaques: preclinical, ex vivo results. IEEE Trans Ultrason Ferroelectr Freq Control.

[63] de Castro JP F, Scott PA, Fransen JW, Demas J, DeMarco PJ, Kaplan HJ, McCall MA (2014). Cone photoreceptors develop normally in the absence of functional rod photoreceptors in a transgenic swine model of retinitis pigmentosa. Invest Ophthalmol Vis Sci.

[64] SoRelle JA, Kanak MA, Itoh T, Horton JM, Naziruddin B, Kane RR (2014). Comparison of surface modification chemistries in mouse, porcine, and human islets. J Biomed Mater Res A.

[65] Yang DL, Cheon SH, Oh CW, Kyung HS (2014). A comparison of the fixation strengths provided by different intraosseous tendon lengths during anterior cruciate ligament reconstruction: a biomechanical study in a porcine tibial model. Clin Orthop Surg.

[66] Li X, He J, Bian W, Li Z, Zhang W, Li D, Snedeker JG (2014). A novel silk-based artificial ligament and tricalcium phosphate/polyether ether ketone anchor for anterior cruciate ligament reconstruction - Safety and efficacy in a porcine model. Acta Biomater.

[67] Seo YJ, Yoo YS, Kim YS, Jang SW, Song SY, Hyun YS, Smolinski P, Fu FH (2014). The effect of notchplasty on tunnel widening in anterior cruciate ligament reconstruction. Arthroscopy.

[68] Larsen MO, Rolin B (2004). Use of the Gottingen minipig as a model of diabetes, with special focus on type 1 diabetes research. ILAR J/Natl Res Council, Inst Lab Anim Resour.

[69] Grehan JF, Hilbert SL, Ferrans VJ, Droel JS, Salerno CT, Bianco RW (2000). Development and evaluation of a swine model to assess the preclinical safety of mechanical heart valves. J Heart Valve Dis.

[70] Badiu CC, Deutsch MA, Bleiziffer S, Krane M, Hettich I, Voss B, Mazzitelli D, Lange R (2014). Early hemodynamic performance of the BioValsalva valved conduit after aortic root replacement. J Heart Valve Dis.

